# Comparison of Existing Clinical Scoring Systems in Predicting Severity and Prognoses of Hyperlipidemic Acute Pancreatitis in Chinese Patients: A Retrospective Study

**DOI:** 10.1097/MD.0000000000000957

**Published:** 2015-06-12

**Authors:** Lei Qiu, Rui Qing Sun, Rong Rong Jia, Xiu Ying Ma, Li Cheng, Mao Chun Tang, Yan Zhao

**Affiliations:** From the Department of Gastroenterology, Shanghai Tenth People's Hospital, Tongji University School of Medicine (LQ, RQS, RRJ, XYM, MCT, YZ); Department of Gastroenterology, Shanghai First People's Hospital of Shanghai, Shanghai JiaoTong University, Shanghai, China (LC).

## Abstract

It is important to identify the severity of acute pancreatitis (AP) in the early course of the disease. Clinical scoring systems may be helpful to predict the prognosis of patients with early AP; however, few analysts have forecast the accuracy of scoring systems for the prognosis in hyperlipidemic acute pancreatitis (HLAP). The purpose of this study was to summarize the clinical characteristics of HLAP and compare the accuracy of conventional scoring systems in predicting the prognosis of HLAP.

This study retrospectively analyzed all consecutively diagnosed AP patients between September 2008 and March 2014. We compared the clinical characteristics between HLAP and nonhyperlipidemic acute pancreatitis. The bedside index for severity of acute pancreatitis (BISAP), Ranson, computed tomography severity index (CTSI), and systemic inflammatory response syndrome (SIRS) scores were applied within 48 hours following admission.

Of 909 AP patients, 129 (14.2%) had HLAP, 20 were classified as severe acute pancreatitis (SAP), 8 had pseudocysts, 9 had pancreatic necrosis, 30 had pleural effusions, 33 had SIRS, 14 had persistent organ failure, and there was 1 death. Among the HLAP patients, the area under curves for BISAP, Ranson, SIRS, and CTSI in predicting SAP were 0.905, 0.938, 0.812, and 0.834, 0.874, 0.726, 0.668, and 0.848 for local complications, and 0.904, 0.917, 0.758, and 0.849 for organ failure, respectively.

HLAP patients were characterized by younger age at onset, higher recurrence rate, and being more prone to pancreatic necrosis, organ failure, and SAP. BISAP, Ranson, SIRS, and CTSI all have accuracy in predicting the prognosis of HLAP patients, but each has different strengths and weaknesses.

## INTRODUCTION

Acute pancreatitis (AP) is a common disease with high morbidity and mortality. The majority of patients with AP (80%–90%) have interstitial edematous pancreatitis, which is a milder form;^1^ however, 10% to 20% of patients develop severe acute pancreatitis (SAP) with mortality reaching 30%.^2^ Thus, it is critical to assess the severity and prognosis of patients with AP early in the disease course, both for triaging patients to appropriate care and designing mechanistic studies for targeted intervention.^3^ The Ranson score plays an important role in the evaluation of severity in AP and has been used clinically for more than 3 decades.^4–6^ Since then, 3 additional clinical scoring systems have been developed (bedside index for severity of acute pancreatitis [BISAP],^7^ computed tomography severity index [CTSI],^4^ and systemic inflammatory response syndrome [SIRS]).^8–10^

It is widely believed that obstruction of the common bile duct by stones (38%) and alcohol abuse (36%) are the most frequent causes of AP.^11,12^ Currently, 12% to 38% of patients with AP result from hyperlipidemia (HL), which has become a well-recognized etiology for AP after gallstone disease and alcohol abuse.^13–15^ Hyperlipidemic acute pancreatitis (HLAP) patients are characterized by the marked hypertriglyceridemia (HTG). Hypercholesterolemia, in contrast to HTG, does not cause AP. Therefore, in a patient with AP, the strongest feature of HLAP is the presence of lipemic serum. Other features that could raise suspicion are pre-existing secondary factors known to cause HTG and family history of HTG. It is generally suggested that serum TG levels above 11.3mmol/L are considered necessary to diagnose HLAP, or the serum TG levels of more than 5.65 to 11.3 mmol/L accompanied by chylous fasting serum after excluding other known risk factors of AP. Compared with other types of pancreatitis, numerous studies have suggested that HTG may result in intensification of AP, more complications, a longer course of disease, and easier relapse;^16–19^ however, some studies have indicated that the clinical course of HLAP does not differ from other forms of pancreatitis.^13^

In the current study, we investigated the difference in clinical characteristics between HLAP and nonhyperlipidemic acute pancreatitis (NHLAP). To identify the strengths and weaknesses of the 4 clinical scoring systems (vide supra) in patients with HLAP, we evaluated the veracity of these scoring systems in categorizing the severity of HLAP. Moreover, we compared the differences between Ranson, BISAP, and CTSI in evaluating the severity of HLAP and NHLAP.

## MATERIALS AND METHODS

After approval by the Ethics Committee of Shanghai Tenth People's Hospital, the present research retrospectively analyzed data of all consecutive patients with AP from Shanghai First People's Hospital and Shanghai Tenth People's Hospital between September 2008 and March 2014. The exclusion criteria included hospitalization discharge with 48 hours, age <18 years, pregnancy, and incomplete information. Data included patient age, gender, diagnosis, admission date, length of hospitalization, relevant laboratory parameters, and radiologic findings.

The diagnosis of AP requires at least 2 of the following 3 features: upper abdominal pain of acute onset, often radiating through to the back; serum amylase and/or lipase levels 3 or more times the upper limit of normal; and findings on cross-sectional abdominal imaging consistent with AP. According to the 2012 Atlanta criteria,^1^ the severity of AP is categorized into 3 levels (mild acute pancreatitis [MAP], moderately severe acute pancreatitis [MSAP], and SAP). MAP lacks organ failure and local or systemic complications. MSAP has transient organ failure (<48 hours), local complications, and/or exacerbation of coexisting disease. SAP is defined by the presence of persistent organ failure (≥48 hours). Organ failure includes pulmonary failure, defined as an arterial PO_2_ <60 mm Hg on room air or the requirement for mechanical ventilation. Cardiovascular failure is defined as the development of shock (systolic pressure <90 mm Hg) that persists after fluid resuscitation. Renal failure is defined as a serum creatinine level >2 mg/dL after rehydration or the need for hemodialysis in patients without pre-existing renal disease. HLAP is diagnosed if the serum TG level reaches 11.3 mmol/L, or the serum TG level of more than 5.65 to 11.3 mmol/L accompanied by chylous fasting serum after excluding other known risk factors of AP.

Each clinical score was calculated based on the worst (most extreme) laboratory measurement obtained on admission and/or within 48 hours of admission. The BISAP and SIRS were calculated within 24 hours of admission, and the Ranson score was recorded within 48 hours of admission. Each patient underwent a contrast-enhanced CT within 48 hours of admission. All CT images were analyzed by 3 professional radiologists. The CTSI score was evaluated after the CT scan within 48 to 72 hours.

All statistical analyses were performed using SPSS 22.0 (SPSS Inc, Chicago, IL). All data were presented as the mean ± SD or a percentage. Normally distributed data were analyzed using a *t* test or Kruskal–Wallis test. Categorical variables were compared using a chi-square test or the Fisher exact test. The sensitivity and specificity of each clinical scoring system were evaluated. A receiver-operating characteristic (ROC) curve is considered the most appropriate statistical tool to describe the performance of continuous variables. The predictive accuracy of each clinical scoring system was measured by the area under the ROC curve (AUC). Pairwise comparisons of the AUC were assessed using the difference between areas, 95% CI, and *P* values. A *P* < 0.05 was accepted as statistically significant.

## RESULTS

### Patient Characteristics

This study enrolled 909 patients with AP; 468 (51.5%) were male and 441 (48.5%) were female. The mean age was 57.0 ± 17.3 years. The etiologies of AP included biliary disease (590 [65%]), alcohol abuse (48 [5.2%]), hypertriglyceridemia (129 [14.2%]), and unknown and idiopathic (142 [15.6%]; Table 1). Seven hundred thirty cases (80.3%) were incipient and 179 cases (19.7%) were recurrent. Eleven patients died during hospitalization. According to the severity classification, 597 (65.7%) were MAP, 234 (25.7%) were MSAP, and 78 (8.6%) were SAP. Twenty six patients had pseudocysts, 31 patients had walled-off necrosis, 206 patients had pleural effusions, and 54 patients developed persistent organ failure (Table 1).

**Table 1:**
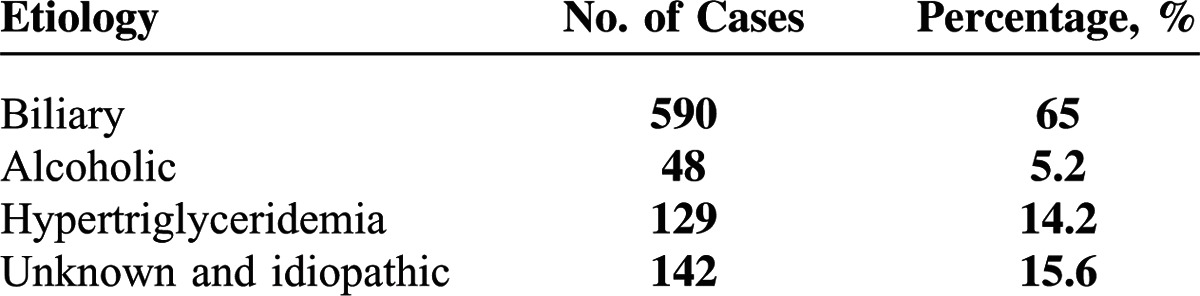
Etiology of Acute Pancreatitis

### HLAP Patient Characteristics

There were 129 patients with HLAP among 909 patients with AP. Seventy six patients (58.9%) were male, with a mean age of 42.8 ± 12.4 years, and 53 patients (40.3%) were female, with a mean age of 49.3 ± 13.2 years. Seventy two patients (57%) had recurrent AP. The number of patients diagnosed with MAP, MSAP, and SAP were 68 (52.7%), 41 (31.8%), and 20 (15.5%), respectively. The number of patients with pseudocysts, pancreatic necrosis, and pleural effusions was 7, 9, and 30, respectively. Fourteen patients developed persistent organ failure. One patient died during the hospitalization (Table 2),

**TABLE 2:**
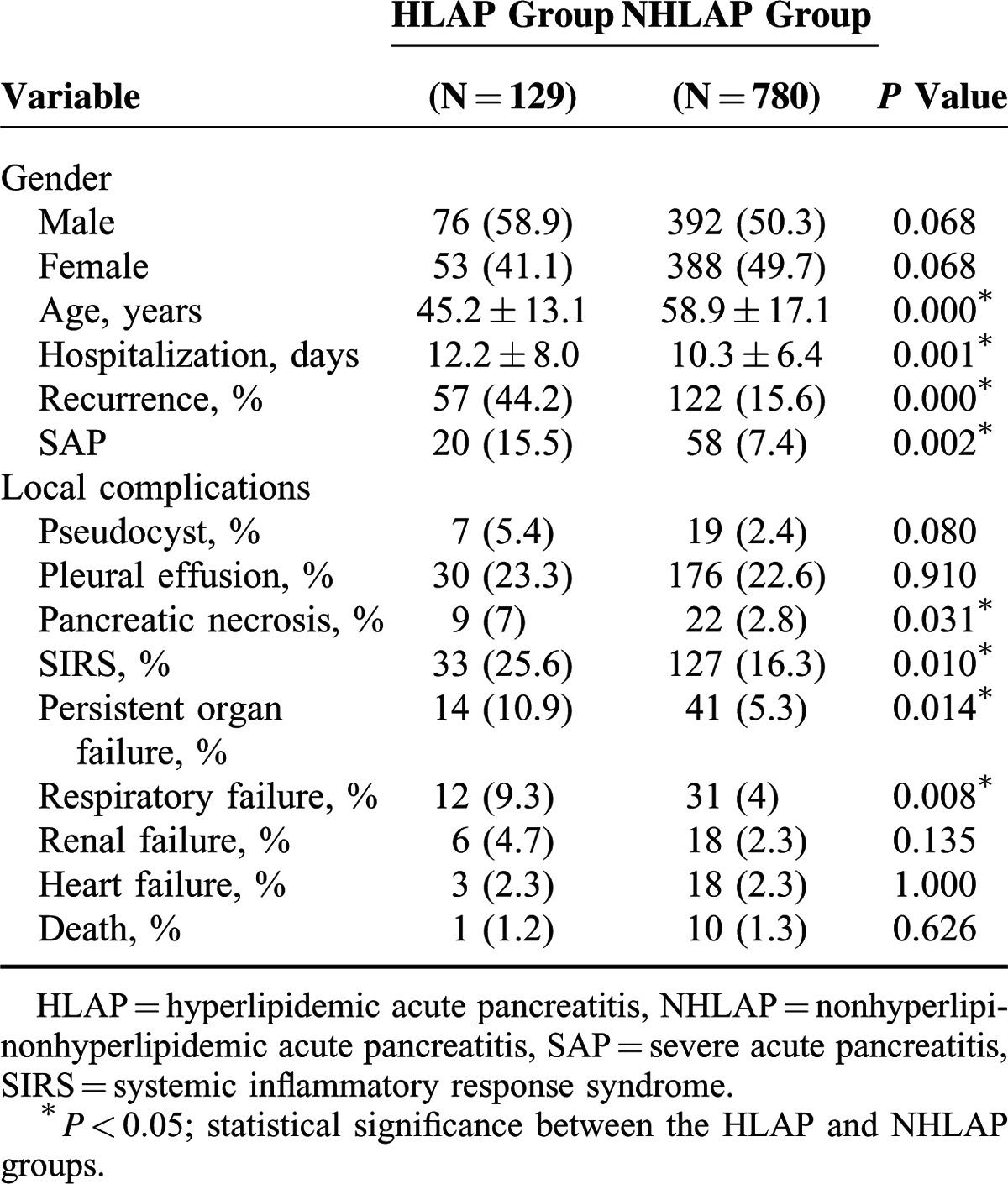
Characteristics of HLAP and NHLAP

### Comparison of Clinical Characteristics Between HLAP and NHLAP

As shown in Table 2, gender, mortality, and local complications (pseudocysts and pleural effusions) did not differ between the HLAP and NHLAP groups; however, the average age of patients with HLAP was significantly lower than patients with NHLAP (45.3 ± 13.1 vs 58.9 ± 17.1 years, *P* < 0.001) and the average length of hospitalization for HLAP patients was longer than NHLAP patients (12.2 ± 8.0 vs 10.3 ± 6.4 days, *P* < 0.001). Moreover, compared to the NHLAP group, patients with HLAP had a higher probability of palindromia (*P* < 0.001), pancreatic necrosis (*P* < 0.005), pulmonary failure (*P* < 0.001), and SAP (*P* < 0.001). The incidence of heart and renal failure was not significantly different between the 2 groups.

### Accuracy of Scoring Systems in Categorizing the Level of AP Severity

As shown in Table 3, the normality test results showed that the score distributions of each clinical scoring system for categorizing the severity of AP obey a positive partial distribution pattern. Therefore, statistical differences were detected by the Kruskal–Wallis test. Each scoring system had a favorable performance in the classification of the level of AP severity.

**TABLE 3:**
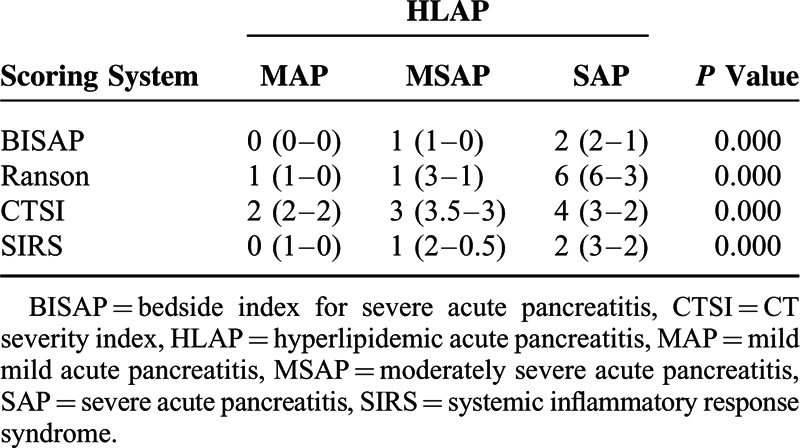
Score Distribution of Each Clinical Scoring System for Categorizing MAP, MSAP, and SAP

### Comparison of Predictive Ability of Scoring Systems in the HLAP Group

**FIGURE 1:**
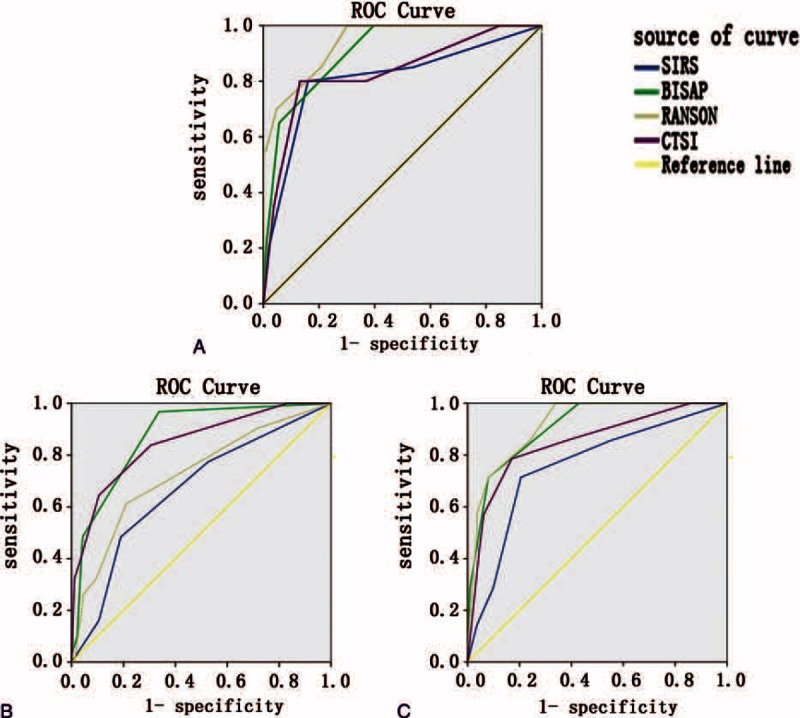
AUC comparison of various scoring systems in predicting SAP (A), local complications (B), and organ failure (C).

Table 4 summarizes the performance characteristics of 4 clinical scoring systems. The AUCs of BISAP, Ranson, SIRS, and CTSI in predicting SAP in the HLAP group were 0.905, 0.938, 0.812, and 0.834, respectively (Figure 1A). The Ranson score had the highest accuracy for predicting SAP in the HLAP group. All the predictions of SAP AUCs with 95% CI >0.5 (*P* < 0.05), and pairwise comparisons of AUC between the 4 scoring systems by Z test showed no statistical differences (*P* > 0.05). Therefore, these 4 scoring systems in predicting SAP in the HLAP group all have accuracy and similar capabilities. The BISAP, Ranson, SIRS, and CTSI cut-offs were 1, 2, 2, and 4, respectively, and the Youden index cut-offs were 0.604, 0.608, 0.640, and 0.668.

**TABLE 4:**
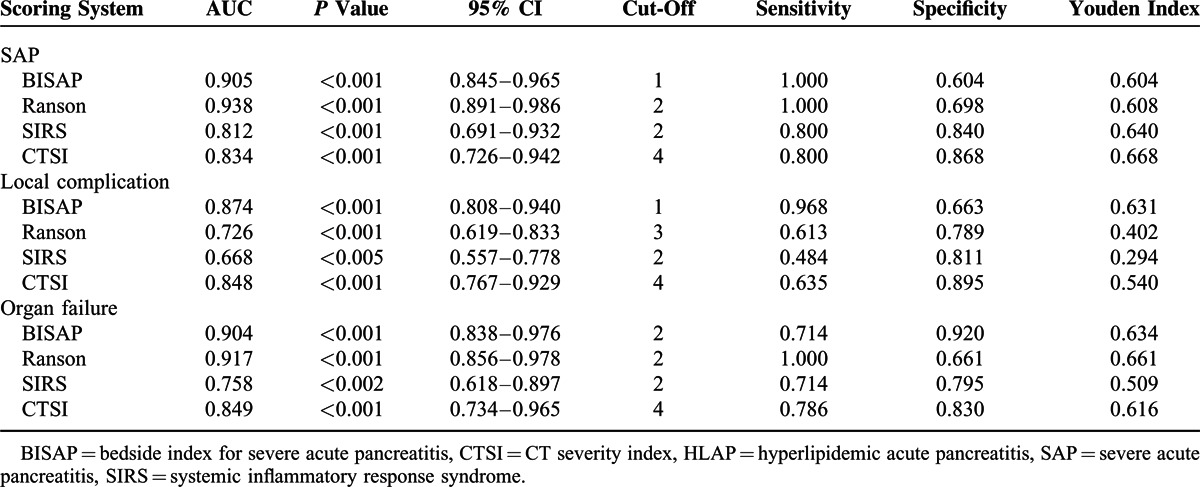
Comparison of Scoring Systems in Predicting SAP, Local Complications, and Organ Failure in the HLAP Group

In predicting local complications in the HLAP group, the AUCs of BISAP, Ranson, SIRS and CTSI were 0.874, 0.726, 0.668, and 0.848, respectively (Figure 1B). The BISAP score showed the highest accuracy for predicting local complications in the HLAP group. Pairwise comparisons of the AUC between the 4 scoring systems revealed that BISAP and CTSI have higher accuracy than SIRS (*P* < 0.05). Therefore, in predicting local complications in the HLAP group, BISAP and CTSI had a better performance. The BISAP, Ranson, SIRS, and CTSI cut-offs were 1, 3, 2, and 4, respectively, and the Youden index cut-offs were 0.631, 0.402, 0.294, and 0.540.

In predicting organ failure in the HLAP group, the AUCs of BISAP, Ranson, SIRS, and CTSI were 0.904, 0.917, 0.758, and 0.849, respectively (Figure 1C). The Ranson score had the highest accuracy for predicting organ failure in the HLAP group. Pairwise comparisons of the AUC between the 4 scoring systems showed the accuracy of Ranson was significantly higher than SIRS (*P* < 0.05). Therefore, in predicting organ failure in the HLAP group, BISAP, Ranson, and CTSI had accuracy and similar capabilities. The BISAP, Ranson, SIRS, and CTSI cut-offs were 2, 2, 2, and 4, respectively, and the Youden index cut-offs were 634, 0.661, 0.509, and 0.616.

### Comparison of Predictive Value of Scoring Systems between HLAP and NHLAP Groups

Table 5 shows the AUCs of the BISAP, Ranson, and CTSI scoring systems in predicting SAP, local complications, pancreatic necrosis, organ failure, and death in the HLAP and NHLAP groups. In prediction of SAP and local complications in NHLAP patients, BISAP had the highest accuracy. Pairwise comparisons of AUC between the 3 scoring systems by the Z test were not statistically different (*P* > 0.05). In HLAP patients, BISAP exhibited a similar predicted performance for SAP and local complications. CTSI demonstrated the maximum predicted AUC for pancreatic necrosis in HLAP and NHLAP patients, and there was no statistically significant difference among the 3 scoring systems (*P* > 0.05). In predicting organ failure and mortality in the NHLAP group, the AUCs of BISAP and Ranson were significantly higher than CTSI (*P* < 0.05). CTSI lacked accuracy in predicting organ failure and mortality in NHLAP patients. In HLAP patients, 3 scoring systems had similar predicted ability for organ failure; however, BISAP showed a lower accuracy in predicting mortality of HLAP patients than Ranson and CTSI.

**TABLE 5:**
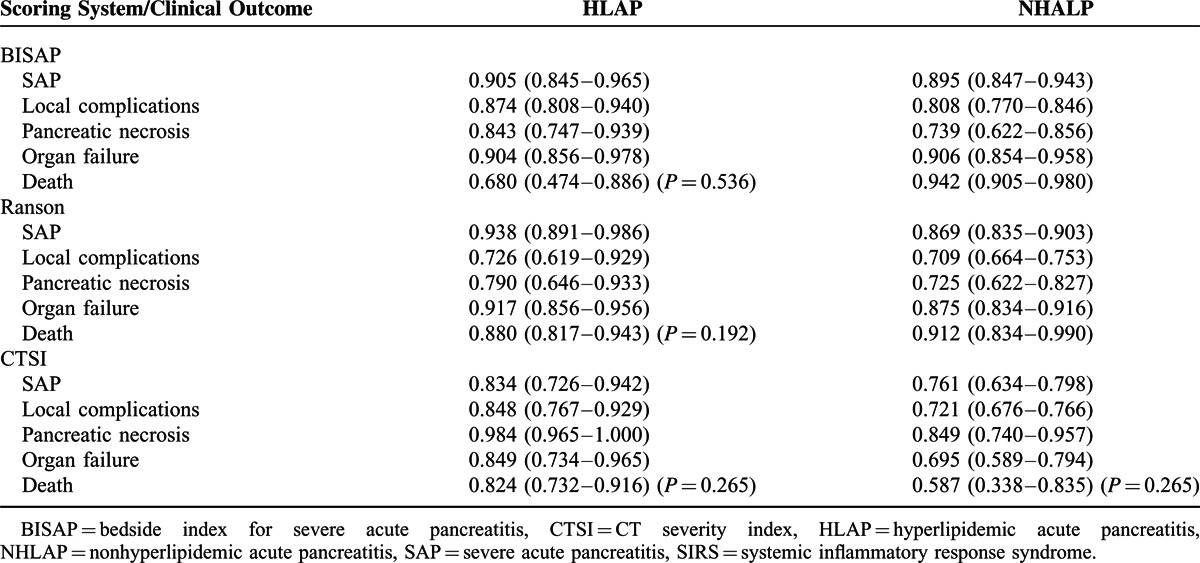
Three Scoring Systems in Predicting the HLAP and NHALP Group Clinical Outcome AUC (95% CI)

## DISCUSSION AND CONCLUSIONS

Since Klastsin first reported hyperlipidemic pancreatitis in 1952, hyperlipidemia has become recognized as a common cause of AP. A multicenter epidemiologic survey showed that the incidence of HLAP is increasing year after year.^20^ In the current study, we summarized the clinical characteristics of patients with HLAP. The present study revealed that the incidence of HLAP was 14.3% in all AP patients. Compared with NHLAP patients, HLAP patients were younger, had longer hospital stays, and higher recurrence rates. Moreover, we found that patients with HLAP were more likely to have pancreatic necrosis and develop organ failure, SAP, and SIRS. Previous studies have suggested that hydrolysis of excessive TG in and around the pancreas by pancreatic lipase lead to accumulation of free fatty acids in high concentrations, and excessive free fatty acids can directly produce acinar cells or capillary injury and cause exorbitant activation of trypsinogen.^21,22^ Increased concentration of chylomicrons in the pancreatic capillaries could cause a disturbance in the pancreatic microcirculation and lead to pancreatic ischemia and necrosis.^21,22^

Based on the clinical characteristics of HLAP, it is necessary to determine the severity of HLAP rapidly and accurately to ensure that potential patients with SAP receive intensive care and adequate treatment as soon as possible. The clinical scoring system has played a significant role in the early prediction of the severity, local complications, and pancreatic necrosis in patients with AP. In the current study, we compared 4 clinical scoring systems in predicting the severity, local complications, and pancreatic necrosis in patients with HLAP.

The Ranson scoring system consists of 11 objective indicators (5 on admission and 6 48 hours after admission), and it is the first scoring system for evaluating the function of early operative intervention in patients with AP. After 40 years of clinical application, the ability of the Ranson scoring system to predict the severity of AP has been widely recognized. A composite score of 3 or more is commonly used to classify a patient with SAP. Khanna et al^23^ reported that the AUCs of the Ranson scoring system in predicting SAP, pancreatic necrosis, and mortality were 0.85, 0.7, and 0.84, respectively, in a study involving 72 AP patients. Forsmark et al^3^ conducted a meta-analysis that included 1307 patients; the analysis indicated that the sensitivity, specificity, PPV, and NPV of the Ranson criteria in predicting SAP were 75%, 77%, 49%, and 91%, respectively. In the current study we found a sensitivity, specificity, and accuracy of 100%, 70%, and 94% using the Ranson criteria for predicting SAP in the HLAP group, 61%, 79%, and 73% for predicting local complications, and 100%, 66%, and 92% for predicting organ failure, respectively. Compared with the other 3 clinical scoring systems, Ranson shows the highest accuracy for predicting SAP and organ failure in patients with HLAP. The cut-off of Ranson in predicting SAP in patients with HLAP was 2, which was lower than general AP patients (cut-off = 3). This difference may be related to the clinical characteristics of HLAP and changes in SAP based on the 2012 Atlanta criteria.^1^ Therefore, we confirmed that the Ranson scoring system also plays a promising role in predicting the severity of patients with HLAP.

The BISAP scoring system, initially proposed by Wu et al,^7^ is a simple and accurate prognostic scoring system containing data that are frequently evaluated on admission. A study found that the accuracy of BISAP in predicting SAP and pancreatic necrosis is 81% and 78%, and the risk of AP patients developing SAP and pancreatic necrosis increased 7.3 and 4.8 times when the BISAP was ≥3, respectively.^24^ In the current study, BISAP had high accuracy in the prediction of SAP, local complications, pancreatic necrosis, and organ failure for patients with HLAP, but poorly predicts the death of HLAP patients. We confirmed that BISAP is an effective tool to predict the prognosis of HLAP patients.

CTSI is a widely used clinical imaging scoring system that has shown a strong positive correlation with the development of complications and mortality in patients with AP.^25,26^ CTSI can observe pancreatic volume, size, contour, and surrounding tissue lesions and clearly display the liquid accumulation and necrosis of the pancreas, which could guide the diagnosis and staging for AP. A study indicated that CTSI can accurately predict prognosis of AP 48 to 72 hours after admission.^27^ The current study results showed that CTSI has higher accuracy in predicting local complications and pancreatic necrosis of HLAP patients. Compared with NHLAP patients, CTSI exerts certain accuracy for predicting the death of HLAP patients.

SIRS was developed to imply the presence of the clinical response to a nonspecific inflammatory insult, which has been validated in a large patient population.^28,29^ Currently, SIRS is not only a clinical symptom, but also a scoring system. SIRS reflects the state of basic vital signs, and further deterioration of SIRS prompts a poor prognosis. Studies have shown that an SIRS score > 2 indicates mortality of AP patients up to 25% within 48 hours after admission.^9^ A prospective study also suggested that elevated SIRS scores within 24 hours after admission indicates increased mortality and risk of persistent organ failure or pancreatic necrosis.^10^ Our study showed that the ability of SIRS to predict SAP, local complications, and organ failure in patients with HLAP is slightly lower than the other 3 scoring systems; however, SIRS still has sufficient accuracy to predict the severity of HLAP, and only contains 4 accessible indicators.

Compared with previous studies of AP patients in western countries, certain difference exists in etiologic distribution. Given to the regional difference, this research probably has more guiding meaning for Chinese patients. Several recent studies take into consideration the complication of multifactorial scoring systems, thus suggesting some of biochemical markers are helpful in earlier prediction and assessment of AP. However, there is no ideal single biochemical marker in assessing the severity of AP. Thus, biochemical markers are not included in our study. In addition, there are still a few disadvantages, including geographic limitations of cases, limited amounts of samples, and sample data bias. Therefore, we need additional multicenter, large-sample studies to compare the reliable predictive ability of conventional scoring systems with respect to the severity of HLAP.

In conclusion, we consider that the clinical symptoms of HLAP patients are worse than those in patients with other causes of AP. We have demonstrated that the currently available 4 scoring systems have good performance on the evaluation of HLAP, and indicate the advantages and disadvantages of each scoring system. We summarize the outcome of this research, and found BISAP has the advantages of simplicity and performed fairly precise ability in the prediction of SAP, local complication, and organ failure. We confirmed that BISAP score system is the first choice to assess the risk stratification and prognostic in patients with HLAP. Additionally, Ranson score system has excellent prediction abilities in both SAP and organ failure; however, its scores are calculated within 48 hours of admission. Consequently, Ranson score system is poor in time effectiveness. For HLAP patients, CTSI score system has outstanding performance in predicting local complication. Comparing with the above 3 score systems, SIRS score system has poor performance in the prediction of SAP, local complication, and organ failure. Therefore, we do not recommend using SIRS to assess the patients with HLAP. We believed that these results are helpful for predicting the severity of HLAP and take effective treatment as soon as possible.
